# Is It Possible to Enhance Adolescents’ Trait Emotional Intelligence Through Service-Learning?

**DOI:** 10.3390/jintelligence14080156

**Published:** 2026-08-01

**Authors:** Francisco D. Fernández-Martín, Patricia Ayllón-Salas, José L. Arco-Tirado

**Affiliations:** Department of Developmental and Educational Psychology, University of Granada, Campus Universitario de Cartuja, s/n, 18071 Granada, Spain; fdfernan@ugr.es (F.D.F.-M.); jlarco@ugr.es (J.L.A.-T.)

**Keywords:** service-learning, secondary school students, competencies, quasi-experimental, intervention

## Abstract

Service-learning interventions have been proposed as a context for examining trait emotional intelligence, a core socioemotional competence associated with cognitive and behavioral outcomes. This pilot quasi-experimental study examined whether participation in a PLACE model (Prepare, Link, Action, Celebrate, Effect) service-learning program was associated with differences in trait emotional intelligence; civic engagement attitudes and behaviors; academic motivation and perseverance; learning mindset; school belonging; dropout intentions; and grade point average among secondary school students. Participants were assigned to an intervention group (*n* = 30) or a control group (*n* = 27). Data were collected using validated instruments, including the Trait Emotional Intelligence Questionnaire, the Civic Engagement Scale, and the Students’ Engagement and Motivation Scale 3.0, along with a single-item measure of dropout intention and official grade point average records. Analyses of covariance, controlling for baseline scores, revealed no statistically significant group differences. Effect sizes showed a mixed pattern, with small effects favoring both groups depending on the outcome. These patterns should be interpreted cautiously, as the study was underpowered to detect small effects. Overall, the findings do not support intervention efficacy but may inform future research with larger samples.

## 1. Introduction

In response to 21st-century labor market demands that emphasize uniquely human skills, such as emotional intelligence, in the context of rapid expansion of artificial intelligence (European Commission, 2020; Organisation for Economic Co-Operation and Development, 2023), service-learning has emerged as a pedagogical approach that integrates academic learning with civic engagement to foster socioemotional and civic competencies in adolescents (Furco & Billig, 2002). However, rigorous quasi-experimental evaluations of service-learning remain scarce, particularly in Spanish secondary education, where it was recently mandated by legislation (State Law 3/2020, of December 29, Which Amends State Law 2/2006, of 3 May 2006, on Education, 2020). Consequently, methodologically robust evidence is still limited (Fernández-Martín & Ayllón-Salas, 2026; Filges et al., 2022).

The present study aims to examine preliminary associations between participation in a service-learning program and outcomes among secondary school students, given the limited research on service-learning in this population, particularly in the Spanish context. Accordingly, this study reports the initial outcomes of the Service-Learning Upscaling Social Inclusion for Kids (SLUSIK) program implemented in Spain, which is designed to enhance students’ instrumental competencies, including social, emotional, academic, and civic competencies.

### 1.1. Theoretical Foundations of Service-Learning

Robert Sigmon was among the first to conceptualize service-learning, defining it as an experiential educational approach grounded in reciprocal learning (Sigmon, 1979). Building on this work, Furco (1996) defined service-learning as a pedagogical method that balances academic learning and community service, ensuring that both providers and recipients benefit.

Service-learning is rooted in the philosophical traditions of Dewey (1938) and Freire (1970). Dewey’s concept of progressive education emphasized learning through active engagement and experience. He argued that experiential learning environments promote lifelong learning and social development while supporting individuals’ personal and educational goals (Dewey, 1938). From this perspective, education should be experiential, connecting prior experiences with new learning to foster competencies necessary for democratic participation (Dewey, 1938; Freire, 1970).

Service-learning is also informed by major learning theories, including Lewin’s (1951) field theory, Vygotsky’s (1978) social constructivism, Lave and Wenger’s (1991) situated learning theory, and Kolb’s (1984) experiential learning model. These frameworks emphasize that learning is optimized through social interaction and the application of knowledge in real-world contexts. Kolb’s (1984) cyclical model—comprising concrete experience, reflective observation, abstract conceptualization, and active experimentation—provides a useful framework for understanding service-learning processes. From a neurobiological perspective, Zull (2002) further suggested that experiential learning engages core brain functions, including acquiring information, interpreting meaning, generating ideas, and applying them.

From a social change perspective, Furco (1996) and Seifer (1998) highlighted the role of service-learning in fostering civic engagement and sustainable social transformation through collaboration among students, educators, and communities.

Service-learning programs are typically implemented during the academic year and integrate community service with academic instruction (Furco & Billig, 2002). They are characterized by three core components: service, reflection, and learning (Centre for European Volunteering, 2021). As a pedagogical approach, service-learning integrates academic and civic competencies to address societal needs and contribute to the public good (Centre for European Volunteering, 2021; European Commission, 2023). It is not an extracurricular activity but an integral component of the curriculum (European Commission, 2023). Its dual aim is to enhance school and community engagement and to strengthen democratic participation by equipping students with relevant civic competencies (European Commission, 2023).

The quality of implementation is essential for achieving meaningful outcomes. Accordingly, adherence to key quality standards is critical, including addressing real community needs; linking activities to the curriculum; promoting meaningful learning and reflection; ensuring active student participation; carefully planning and designing activities; providing sufficient duration and intensity; monitoring progress; fostering community partnerships; and recognizing student contributions (European Commission, 2023; Filges et al., 2022). Additionally, adapting service-learning to diverse contexts ensures that students and community partners are actively involved in program design and implementation (Filges et al., 2022).

### 1.2. Service-Learning Programs in Secondary Education

Farber and Bishop (2018) reported that, although numerous service-learning initiatives have been developed, fewer than 9% are designed for secondary education students. Thus, within secondary education, service-learning programs remain the exception rather than the norm (Farber & Bishop, 2018). This does not imply that such programs are absent at this level; rather, it suggests that data collection methods used with this population may not effectively capture program outcomes (Centre for European Volunteering, 2021). In addition, variability in implementation has contributed to inconsistencies in reported student outcomes across the literature (Billig, 2000; Farber & Bishop, 2018).

In Spain, service-learning was formally incorporated into secondary education through State Law 3/2020, of December 29, Which Amends State Law 2/2006, of 3 May 2006, on Education (2020). This policy development has led to an increase in agreements and regional initiatives, resulting in the expansion of service-learning programs across autonomous communities (Batlle et al., 2019).

Spain represents a distinctive European context, as service-learning has only recently been integrated into the secondary education curriculum through national legislation (State Law 3/2020, of December 29, Which Amends State Law 2/2006, of 3 May 2006, on Education, 2020). This framework promotes decentralized implementation across 17 autonomous communities with diverse educational policies. Nevertheless, no Spanish studies on service-learning effectiveness met the methodological quality standards identified in the most recent meta-analysis by Filges et al. (2022), highlighting a significant evidence gap that the present study seeks to address.

### 1.3. Service-Learning Outcomes

Service-learning programs have the potential to enhance academic achievement and support the development of competencies by providing students with opportunities to apply their knowledge in authentic community contexts (Furco, 2010). Accordingly, service-learning has been associated with a range of outcomes, including social and emotional competencies (i.e., skills that enable the expression, regulation, and understanding of thoughts, emotions, and behaviors in everyday interactions; Schoon, 2021), civic competencies (i.e., the capacity to act as responsible citizens and engage actively in civic and social issues; European Commission, 2019), motivational outcomes (i.e., the degree of engagement in the teaching and learning process; Bridgeland, 2010), and academic outcomes (i.e., changes in cognitive processes and academic performance, including the acquisition and application of knowledge; Conway et al., 2009).

First, service-learning has been shown to promote civic engagement by encouraging sustained participation in social and public issues (Flores, 2018). These programs expose students to social challenges and to the institutions responsible for addressing them (Flores, 2018; Meyers, 2009). In addition, service-learning can foster students’ interest in taking active roles in their communities, supporting the development of political and social awareness. Consequently, service-learning holds strong potential for fostering engaged citizenship (Flores, 2018). For example, Kahne and Sporte (2008) found that classroom opportunities to discuss social problems and potential solutions were associated with higher levels of civic engagement.

Second, service-learning connects academic content to real-world issues and encourages active problem solving, making it a potentially effective approach for enhancing student engagement and motivation (Bridgeland et al., 2008; Marttinen et al., 2019). Empirical evidence suggests that participation in service-learning is associated with increased interest in academic subjects (Farber & Bishop, 2018). Additionally, students who participate in service-learning programs have been found to exhibit higher levels of motivation (Furco, 2010; Melchior, 1995, 1998). These students also demonstrate higher rates of class attendance compared with their peers (Melchior, 1998; Scales et al., 2006). Consequently, they tend to show greater interest in and commitment to their educational institutions than students who do not participate in service-learning programs (Chiva-Bartoll et al., 2020; Furco, 2010; Melchior, 1995, 1998).

Third, the effects of service-learning extend beyond the classroom. Secondary education is a critical stage, as academic performance during this period can influence whether students complete their education (Organisation for Economic Co-Operation and Development, 2023). Interventions delivered during school hours are particularly important for reaching students at risk of dropping out or experiencing academic difficulties (Chung & McBride, 2015).

Evidence suggests that students who participate in service-learning programs tend to exert greater effort to achieve strong academic outcomes than their peers who do not participate (Furco, 2010; Scales et al., 2000). As a result, they may demonstrate higher academic performance, as reflected in grades and grade point average (Fraley, 2015). In addition, students participating in service-learning report enhanced learning outcomes in these courses compared with traditional academic classes (Capella-Peris et al., 2019).

These factors are directly related to dropout risk, with service-learning participants showing a lower likelihood of leaving school prematurely (Bridgeland et al., 2006; Fraley, 2015). Some studies further suggest that participation in service-learning may increase the likelihood of graduation (McFarland, 2014).

Finally, service-learning provides a distinctive opportunity to apply social and emotional competencies in authentic contexts. Research indicates that the adolescent brain is particularly sensitive to environmental influences (Chung & McBride, 2015). During this developmental stage, experience-dependent cognitive and behavioral activities can shape brain structure and functioning (Burnett et al., 2011). Accordingly, practicing skills in early adolescence—such as through participation in service-learning—may have long-term effects extending into adulthood (Chung & McBride, 2015). Although research on the impact of service-learning on social and emotional competencies remains limited (Chiva-Bartoll et al., 2020), emerging evidence highlights its potential to transform the teaching–learning process by engaging students in real-world social problems and enhancing their understanding (Fernández-Bustos et al., 2024).

### 1.4. Impact of Service-Learning on Trait Emotional Intelligence

Trait emotional intelligence, also referred to as trait emotional self-efficacy, is defined as a constellation of emotional self-perceptions located at the lower levels of personality hierarchies (Petrides, 2010; Petrides & Mavroveli, 2020). Unlike ability-based models of emotional intelligence, this perspective conceptualizes the construct as individuals’ perceptions and beliefs about their own emotional capabilities (Petrides et al., 2016; Petrides & Mavroveli, 2020). Trait emotional intelligence is considered a core socioemotional competence that contributes to adaptive functioning across the lifespan (Özal et al., 2024; Petrides et al., 2016). Although conceptualized as a personality-like disposition with relative cross-situational stability (Petrides, 2009), meta-analytic evidence suggests that it remains malleable during adolescence (Roberts et al., 2006), thereby allowing for potential intervention effects through experiential learning approaches (Kotsou et al., 2019).

Service-learning programs in higher education have been associated with positive socioemotional outcomes, including higher emotional intelligence. For example, business students participating in a six-month program showed improvements in self-emotion appraisal, regulation, and use (Pong & Lam, 2023). Similarly, medical students demonstrated gains in both intrapersonal and interpersonal emotional competencies (Martinez, 2021), and pre-service teachers reported enhanced self-awareness and empathy (Byrne, 2009).

Associations between service-learning and emotional development may be particularly pronounced among older students in secondary education, given the higher levels of critical thinking and coordination required (Hanna & Treece, 2014). Additionally, Hanna and Treece (2014) found that female high school students tended to show greater gains in emotional development from service-learning experiences than their male peers.

Despite evidence supporting the relationship between service-learning and emotional intelligence, important gaps remain, particularly in secondary education. While a substantial body of research exists in higher education, the high school context remains comparatively underexplored for the development of trait emotional intelligence. Moreover, there is a lack of rigorous quasi-experimental studies examining these associations in adolescents (Hanna & Treece, 2014). Current research is also limited by restricted geographic diversity, highlighting the need for further studies in non-United States contexts (Fernández-Martín & Ayllón-Salas, 2026).

### 1.5. This Study

Despite the widespread implementation of service-learning programs worldwide, the literature highlights the need for rigorous studies using experimental or quasi-experimental designs in secondary education (Fernández-Martín & Ayllón-Salas, 2026; Filges et al., 2022). For example, the meta-analysis by Filges et al. (2022) reported inconclusive evidence from studies conducted in the United States. Similarly, a systematic review by Fernández-Martín and Ayllón-Salas (2026) identified mixed and inconsistent effects in non-United States contexts. Although some studies report small-to-moderate positive effects on academic and noncognitive outcomes, the magnitude and consistency of these effects vary considerably across study designs and contexts, limiting firm causal inferences. Notably, trait emotional intelligence among Spanish adolescents remains underexamined, as most evaluations focus on broader socioemotional constructs rather than this specific construct (Fernández-Martín & Ayllón-Salas, 2026). These limitations highlight the need for rigorously designed and implemented service-learning studies in Spain, particularly those employing quasi-experimental designs with control groups, high implementation fidelity, and comprehensive assessment of trait emotional intelligence and related noncognitive outcomes.

Accordingly, the present study examines preliminary associations between participation in a service-learning program and several outcomes among Spanish secondary school students: trait emotional intelligence, civic competence, academic motivation and engagement, dropout intention, and grade point average. Given the pilot nature of the study, the primary hypothesis focused on trait emotional intelligence as the main outcome. It was hypothesized that students in the intervention group would report higher levels of trait emotional intelligence than those in the control group. The remaining variables (i.e., civic attitudes and behaviors, academic motivation and engagement, dropout intention, and grade point average) were examined as exploratory outcomes.

## 2. Materials and Methods

### 2.1. Participants

The sample comprised 57 first-year secondary school students from a public school located in an urban area of Granada, Spain. The mean age was 12.9 years (*SD* = 0.44). The sample included 38 male students (66.7%) and 19 female students (33.3%). The intervention group consisted of 30 students (age: *M* = 12.80, *SD* = 0.50; 56.67% male), whereas the control group comprised 27 students (age: *M* = 13.00, *SD* = 0.34; 77.78% male).

A nonprobability convenience sampling technique was used (Kalton, 2020). The sampling procedure involved three steps: (a) several secondary schools were invited to participate, and one school agreed to take part in the study; (b) teachers from the participating school were invited to volunteer to implement the program with their students; and (c) after obtaining institutional approval and parental consent, four teachers and their 62 students from two comparable first-year classroom groups enrolled in the program.

### 2.2. Materials

Sociodemographic Questionnaire. A self-report questionnaire was developed for this study to collect basic sociodemographic information. It consisted of three items assessing age, gender, and place of residence. The residence variable included two response options: city and village.

Trait Emotional Intelligence Questionnaire—Spanish Version (Pérez-González, 2003). The Spanish adaptation of the Trait Emotional Intelligence Questionnaire is a Likert-type scale comprising 30 items distributed across 15 facets, with responses ranging from 1 (*completely disagree*) to 7 (*completely agree*). Consistent with the original version (Petrides, 2009), the instrument evaluates four higher-order trait emotional intelligence factors and yields a global trait emotional intelligence score: (a) wellbeing (i.e., facets of trait optimism, trait happiness, and self-esteem); (b) self-control (i.e., tendency to manage emotions and impulses through the facets of emotion regulation, impulsiveness, and stress management); (c) emotionality (i.e., ability to perceive and express emotions via the facets of trait empathy, emotion perception, emotion expression, and relationship skills); and (d) sociability (i.e., affective functioning in interpersonal contexts and comprising the facets of assertiveness, emotional management of others, social relationships, and social competence). Regarding its psychometric properties, the original version demonstrated satisfactory reliability, with overall Cronbach’s alpha (α) of 0.89 for females and 0.92 for males (Petrides, 2009), and validity (i.e., factor analysis demonstrating its factorial structure) (Laborde et al., 2016). In this study, the questionnaire exhibited a α of 0.86 and a McDonald’s omega (ω) of 0.87 for the overall scale in the pre-test and a α of 0.84 and a ω of 0.85 in the post-test.

Civic Engagement Scale—Spanish Version (Ayllón-Salas et al., 2025). The Spanish adaptation of the Civic Engagement Scale follows the structure of the original instrument (Doolittle & Faul, 2013) and consists of 14 items rated on a 7-point Likert scale (1 = *disagree/never*, 7 = *agree/always*). The scale assesses two dimensions of civic engagement: (a) civic attitudes (i.e., individuals’ personal beliefs and feelings regarding their involvement in the community, as well as their perceived capacity to contribute to social change); and (b) civic behaviors (i.e., concrete actions through which individuals actively participate and make a difference in their community). The Spanish version demonstrated satisfactory structural validity and internal consistency across both subscales (α = 0.91 for civic attitudes and α = 0.87 for civic behaviors). In the present study, internal consistency was acceptable. For the civic behavior subscale, Cronbach’s α = 0.76 and McDonald’s ω = 0.77 at pretest, and Cronbach’s α = 0.80 and McDonald’s ω = 0.80 at posttest. For the civic attitudes subscale, Cronbach’s α = 0.82 and McDonald’s ω = 0.83 at pretest, and Cronbach’s α = 0.81 and McDonald’s ω = 0.82 at posttest.

Students’ Engagement and Motivation Scale (The Roadmap Project, 2015). This instrument was originally developed by the Youth Development for Education Results work group of the Road Map Project, under the coordination of the Youth Development Executives of King County. It comprises 43 items rated on a Likert scale ranging from 1 (strongly disagree) to 5 (strongly agree), together with 10 multiple-choice questions. This instrument is intended to broaden the understanding of the factors associated with students’ academic success by integrating both psychometric and practical information. The scale encompasses three subscales: (a) academic perseverance (i.e., the disposition to exert sustained effort and assume responsibility for one’s own academic progress); (b) learning mindsets (i.e., the belief in one’s capacity to learn and achieve, the perception that intelligence is malleable through individual effort, the enjoyment of learning and openness to new challenges, and the perceived relevance of school-based tasks for longer-term personal goals); and (c) school belonging (i.e., students’ sense of acceptance and support within the school learning community). The original version demonstrated adequate levels of validity and internal consistency (i.e., α between 0.67 and 0.84) (The Roadmap Project, 2015). Students’ Engagement and Motivation Scale was culturally adapted for Spanish adolescents during SLUSIK project development (Brozmanová-Gregorová et al., 2024). Validation across 12 European countries (*n* > 2000 secondary students) confirmed adequate psychometric properties. Indeed, in this study, the scale yielded acceptable levels of internal consistency (α = 0.87 and ω = 0.88 in the pre-test, and α = 0.82 and ω = 0.83 in the post-test).

School Dropout Intention. School dropout intention was assessed using a single-item measure (“I am thinking about leaving this school”), rated on a 5-point Likert scale (1 = *completely disagree*, 5 = *completely agree*).

Academic Report. Academic performance was operationalized as grade point average, calculated from official academic records provided by the participating school before and after the intervention. Grade point average was computed as the mean of students’ grades across all subjects, with possible scores ranging from 0 to 10.

### 2.3. Design and Procedure

This study was conducted in accordance with the ethical standards of the Declaration of Helsinki (World Medical Association, 2013) and was approved by the Ethics Committee of the University of Granada (1974/CEIH/2021).

The study employed a cluster quasi-experimental controlled design. Following the provision of informed consent, classroom groups were randomly assigned to either the intervention or control condition to minimize potential sources of bias. A minimum sample size of 30 participants per group was considered desirable to maintain the robustness of *t* tests under potential violations of normality (Bloom, 2003). However, attrition occurred in both groups, with two participants lost from the intervention group and three from the control group. Students in the control group continued their regular school schedule and received standard instruction across all subjects. They completed typical curricular activities planned by their teachers and did not participate in service-learning experiences or engage with community partners.

The service-learning program implemented in this study was based on the PLACE model, an intervention framework developed within the Erasmus+ SLUSIK project to facilitate the integration of service-learning into secondary education and support teachers in its implementation (Centre for European Volunteering, 2022). The model builds on evidence-informed practices aimed at promoting students’ competencies and academic performance.

The PLACE service-learning model is structured into five sequential stages: (a) *prepare*, which involves training teachers and preparing the service-learning activities; (b) *link*, which connects students with community partners and role models; (c) *action*, which engages students in addressing real-world issues; (d) *celebrate*, which involves presenting students’ achievements in collaboration with community partners; and (e) *effect*, which reflects the reciprocal benefits and the meaningful contributions made by students (Centre for European Volunteering, 2022).

The PLACE service-learning model incorporates several key features. First, it employs nonformal educational methodologies grounded in experiential, learning-by-doing approaches (e.g., simulation exercises, games, outdoor activities, expressive activities, debriefing, and reflection). Second, it promotes collaboration with universities through the involvement of role models (i.e., university students with prior experience in service-learning who support secondary school students in authentic learning contexts). Third, it emphasizes the development of cross-sector partnerships at the local level through community partners (i.e., nonprofit organizations that provide opportunities to address real community needs in authentic contexts; Centre for European Volunteering, 2022). Additional details on the model are available in the Centre for European Volunteering (2022).

The intervention was grounded in a trait emotional intelligence framework, based on the premise that emotional competencies can be enhanced through structured, experiential, and reflective learning. Accordingly, each component of the program was designed to target specific dimensions of trait emotional intelligence. Activities focused on emotional perception aimed to improve participants’ ability to identify and interpret their own emotions and those of others. Tasks centered on emotion regulation were intended to foster strategies for managing emotional responses in challenging situations. Exercises promoting empathy sought to enhance perspective-taking and sensitivity to others’ emotional states, while activities related to self-control aimed to strengthen impulse regulation and behavioral moderation. Finally, sociability was addressed through collaborative tasks and guided interaction, with the purpose of improving communication, assertiveness, and positive social engagement. Building on this rationale, the intervention was designed to foster trait emotional intelligence through two structured service-learning experiences aligned with its core dimensions.

The implementation of the service-learning PLACE model lasted for 7 months and consisted of five stages. The first stage (i.e., prepare) involved training secondary school teachers to implement the service-learning model and university students to act as role models, as well as designing and planning the service-learning framework. The secondary school teachers’ training seminar aimed to enhance secondary school teachers’ knowledge and skills to implement the service-learning PLACE model. Thus, a four-hour session training was delivered using an active and participatory methodology to cover the following contents: (a) introduction of the program staff, participants, and training plan; (b) justification of the program; (c) introduction to service-learning: non-formal learning, the five stages of the service-learning PLACE model, and the core actors in the service-learning PLACE model (i.e., secondary school teacher, secondary school student, community partner, role model, and program staff); (d) implementation of the service-learning PLACE model; (e) description and use of the service-learning PLACE model Toolkit (i.e., study protocol, a set of materials in which the PLACE service-learning model was presented in a structured manner: task to be done by the teacher, suggested tools to be used, and outcomes expected in each of the stages of the PLACE model) (Centre for European Volunteering, 2022); and (f) analysis of potential conflicts or unpredicted situations.

Concurrently with the training of secondary school teachers, a recruitment and preparation process was conducted for university students who would serve as role models during the implementation of the service-learning PLACE model. The program was disseminated among undergraduate students enrolled in Social Education and Primary Education teacher training programs. These students were informed about the program’s characteristics, requirements, tasks, and potential benefits and were invited to participate. Ultimately, six university students with prior service-learning experience joined the program as role models.

The role model training seminar was designed to strengthen the knowledge, attitudes, and skills necessary to establish effective and safe mentoring relationships, with an emphasis on culturally sensitive communication and practices. To this end, a 4 h session was delivered using an active and participatory methodology. The training covered the following components: (a) introduction to program staff, participants, and the training plan; (b) program requirements; (c) clarification of personal goals and expectations for the mentoring relationship; (d) roles and responsibilities of participants; (e) strategies for initiating mentoring relationships; (f) approaches to relationship development and sustainability; (g) ethical and safety considerations; (h) available support resources for role models; (i) opportunities and challenges associated with mentoring specific youth populations; and (j) procedures for session evaluation and record keeping.

Finally, two service-learning experiences were collaboratively designed and planned by secondary school teachers and university program staff over four sessions. During this process, potential community partners were also identified. Service-learning experiences focused on (a) environmental sustainability and (b) civic engagement. In the environmental sustainability project, students collaborated with a local nongovernmental organization to address climate and environmental stewardship within and beyond the school context. Activities included systematic waste mapping in nearby parks followed by community clean-up actions; the design of awareness-raising materials to reduce single-use plastics; the creation and maintenance of a school vegetable garden, including planting seasonal crops and applying sustainable cultivation techniques; and participation in a supervised reforestation initiative in a nearby natural area, where students planted native trees as part of a broader ecological restoration effort. In the civic engagement project, students partnered with a neighborhood association to conduct brief interviews with residents to identify barriers to participation and local priorities. Based on these findings, students developed low-threshold civic micro-actions (e.g., the distribution of informational leaflets), produced communication materials to promote respectful use of shared public spaces, and organized a youth–neighborhood dialogue session to discuss community challenges and co-create potential solutions.

The *link* stage focused on connecting key stakeholders, including secondary school students, community partners, and role models. This stage involved identifying community needs and selecting activities aligned with curricular learning objectives, as well as establishing a shared understanding of each stakeholder’s role. It was implemented over two sessions. During this phase, project teams finalized the design of the school garden and reforestation logistics in collaboration with the environmental nongovernmental organization (e.g., species selection, site preparation, and safety protocols). They also agreed on interview guides, participant recruitment strategies, and the agenda for the dialogue session with the neighborhood association. The transition to the next stage occurred during the final session of the *prepare* phase, facilitating the introduction of role models to secondary school students.

The *action* stage was implemented across 20 sessions conducted weekly or twice weekly. This stage emphasized the integration of classroom learning with real-world issues and promoted active student engagement with community partners. It provided students with opportunities to apply their knowledge to authentic challenges, fostering responsibility and civic engagement within and beyond the school community. Reflection on experiences and outcomes was embedded throughout this phase. Activities were carried out in collaboration with community partners. In the environmental project, tasks included waste mapping and clean-up activities, installation and maintenance of the school garden, and participation in a field day focused on native tree planting at a restoration site. In the civic project, activities included rapid needs assessments through brief interviews, the design and implementation of civic micro-actions, the production of informational posters and digital materials, and the facilitation of a youth–neighborhood dialogue session held at the school. Structured reflection activities, guided by teachers and university role models, were integrated throughout to link experiences with curricular objectives in Biology, Geography, Civic and Ethical Education, and Language and Literature.

The *celebrate* stage was conducted over three sessions. During this phase, students presented their learning and achievements in collaboration with community partners. A public event was organized, open to community members, including families, friends, and local stakeholders. The event featured a school-wide exhibition with posters, photographs, and brief presentations documenting the school garden development and initial crops, waste-mapping findings and clean-up outcomes, the reforestation activities and species planted, and the key conclusions and commitments derived from the youth–neighborhood dialogue.

The *effect* stage, the final phase of the intervention, aimed to support students in evaluating their service-learning experiences and producing formal reports. During this stage, students consolidated their ideas and applied their learning, resulting in reciprocal benefits for both students and the community. This phase was implemented over four sessions. Students produced individual and group reflective reports addressing perceived community impact and personal learning. They also participated in feedback meetings with community partners to discuss strengths and areas for improvement and identified feasible strategies for sustaining project outcomes (e.g., establishing a maintenance rota for the school garden, scheduling periodic visits to the reforestation site with the nongovernmental organization, and organizing recurring neighborhood dialogue sessions). Guided activities explicitly connected these experiences to longer-term civic responsibility, school belonging, and socioemotional skill development.

In parallel with program implementation, a monitoring plan was established to ensure intervention fidelity. This plan included biweekly follow-up meetings with secondary school teachers, community partners, and role models to monitor implementation processes and maintain consistency.

Finally, outcome measures were collected before and after the implementation of the service-learning PLACE model to assess potential statistically and educationally significant effects.

### 2.4. Data Analysis

No a priori power analysis was conducted because this pilot study aimed to estimate effect sizes and variability for service-learning interventions targeting trait emotional intelligence in the Spanish context, where prior effect size estimates are unavailable (Fernández-Martín & Ayllón-Salas, 2026; Filges et al., 2022). A post hoc power analysis conducted using G*Power 3.1 indicated 54% power to detect moderate effects (*d* = 0.40, α = 0.00625, Bonferroni-corrected for eight outcomes) with a sample size of *N* = 57. The observed effect size for the primary outcome, trait emotional intelligence (*d* = 0.51, *p* = .11), exceeded commonly suggested pilot study progression criteria (*d* > 0.30), providing preliminary support for conducting a full-scale randomized controlled trial (Cocks & Torgerson, 2013).

Preliminary analyses were conducted following the recommendations of Tabachnick and Fidell (2021). These included the computation of descriptive statistics and examination of data distribution (i.e., skewness, kurtosis, and Mardia’s coefficients of multivariate skewness and kurtosis). Assumptions of linearity, as well as the presence of outliers, missing data, and influential cases, were also assessed. Prior to conducting analyses of covariance (ANCOVAs), key assumptions were tested. Homogeneity of variances was confirmed using Levene’s test (*p* > .05), and homogeneity of regression slopes was supported by non-significant Group × Pretest interactions across all outcome variables. Internal consistency of the measures was evaluated using Cronbach’s α and McDonald’s ω, with values of 0.70 or higher considered acceptable (Green, 2015).

Baseline equivalence between groups was examined by calculating Cohen’s *d* effect sizes for each outcome at pretest. This analysis was conducted to assess whether preintervention differences systematically distinguished the two groups.

Prior to the main analyses, attrition was examined to assess potential bias. Independent-samples *t* tests and chi-square tests were conducted to compare completers (*n* = 57) and dropouts (*n* = 5) on baseline characteristics, including gender, χ^2^(1) = 0.12, *p* = .73; age, *t*(60) = 0.89, *p* = .38; and trait emotional intelligence, *t*(60) = 0.98, *p* = .33. No statistically significant differences were found, suggesting non-differential attrition. A sensitivity analysis using a baseline observation carried forward approach confirmed the robustness of the findings. Accordingly, analyses based on completers (*n* = 57) are reported as primary.

For each outcome, ANCOVAs were conducted with posttest scores as the dependent variables, pretest scores as covariates, and group as a fixed factor. Due to between-group differences in gender distribution, gender was also included as a covariate in all models. Exploratory analyses examined the Group × Gender interaction; however, these analyses were interpreted cautiously given the limited statistical power. Effect sizes were estimated using partial η^2^. Bonferroni-adjusted post hoc comparisons were performed, and Cohen’s *d* was calculated based on estimated marginal means and the pooled observed standard deviation.

Because the study included only one classroom per condition, classroom and treatment effects were fully confounded. Although the inclusion of pretest scores and gender as covariates improves precision and partially accounts for observed differences, unobserved classroom-level influences cannot be ruled out. Therefore, the results are interpreted as adjusted associations consistent with a pilot feasibility design rather than as definitive causal estimates.

All analyses were conducted using jamovi version 2.7.31 (The Jamovi Project, 2026).

## 3. Results

The analysis of skewness and kurtosis suggested that the baseline distributions were broadly comparable across groups (see Appendix A for details). Baseline comparability between groups was examined descriptively using Cohen’s *d* effect sizes for pretest scores. The results indicated generally small differences across most outcome variables; however, global trait emotional intelligence showed a relatively larger pretest difference (see Table 1). Given the pilot nature of the study, these effect sizes are reported solely to describe the magnitude and direction of preintervention differences and should not be interpreted as evidence of group equivalence or nonequivalence.

Table 2 presents the ANCOVA results for all outcome variables after adjusting for pretest scores and gender. Across all variables—global trait emotional intelligence, civic attitudes, civic behavior, academic perseverance, learning mindset, school belonging, school dropout intention, and grade point average—the group effect was not statistically significant (all *p* > .05).

Effect sizes indicated that the magnitude of group differences was consistently small across outcomes, with partial η^2^ values ranging from 0.00 to 0.06. These effect sizes are reported as descriptive indices to contextualize the non-significant inferential findings.

Gender did not have a statistically significant main effect on any outcome (all *ps* > .05). Exploratory analyses further indicated that the Group × Gender interaction was not statistically significant for any variable (all *ps* > .05), suggesting that the intervention did not differentially affect male and female students.

It should be noted that Bonferroni-adjusted post hoc comparisons were based on unadjusted posttest means and therefore did not control for baseline differences. Consequently, interpretations of group effects rely primarily on the ANCOVA-adjusted estimates rather than on post hoc contrasts.

Bonferroni-adjusted post hoc comparison results are presented in Table 3. Overall, differences between the adjusted means of the intervention and control groups were small across all variables, and none reached statistical significance: global trait emotional intelligence (*M* difference = 0.19, *p* > .05), civic attitudes (*M* difference = 0.15, *p* > .05), civic behavior (*M* difference = −0.43, *p* > .05), academic perseverance (*M* difference = 0.05, *p* > .05), learning mindset (*M* difference = −0.20, *p* > .05), school belonging (*M* difference = 0.22, *p* > .05), dropout intention (*M* difference = −0.22, *p* > .05), and grade point average (*M* difference = −0.14, *p* > .05).

Cohen’s *d* values are reported for descriptive purposes only. Given the pilot nature of the study and the absence of statistically significant findings, these estimates should not be interpreted as evidence of practical significance or intervention efficacy.

## 4. Discussion

The present study aimed to examine preliminary associations between participation in a service-learning program and secondary school students’ trait emotional intelligence, civic competence, academic motivation and engagement, dropout intention, and grade point average.

First, the primary hypothesis proposed that students in the intervention group would exhibit higher levels of trait emotional intelligence than those in the control group. Although the ANCOVAs indicated a strong association between pretest scores and posttest outcomes, the between-group difference was not statistically significant. Descriptively, the observed difference was small; therefore, the primary hypothesis was not supported.

Regarding the exploratory outcomes, no statistically significant between-group differences were observed for civic attitudes, civic behavior, academic motivation and engagement, dropout intention, or grade point average. Overall, adjusted differences were small and inconsistent across variables. For civic behavior, the control group showed slightly higher scores; however, this difference was not statistically significant. Taken together, these results do not provide clear evidence of differential change associated with the program.

The absence of statistically significant group differences may be partially explained by the use of a conservative Bonferroni correction and the limited sample size, both of which increase the likelihood of Type II error (Armstrong, 2014). Consequently, the statistical power of the study may have been insufficient to detect small effects. Because Bonferroni-adjusted post hoc comparisons are based on unadjusted posttest means, they do not account for baseline differences; thus, these contrasts should be interpreted cautiously. Accordingly, ANCOVA-adjusted effect sizes are emphasized as descriptive indicators of between-group differences that account for pretest scores and gender.

Although no statistically significant effects were detected, the findings provide preliminary information relevant to feasibility in a pilot context. Any interpretation of the results as “promising” should be restricted to descriptive patterns, specifically the small ANCOVA-adjusted effect sizes observed for trait emotional intelligence, civic attitudes, and school belonging. These findings should be considered preliminary and interpreted cautiously, underscoring the need for further investigation using larger samples and more rigorous multi-classroom and multisite designs rather than as evidence of intervention effectiveness.

Finally, beyond statistical significance, the reported ANCOVA-adjusted effect sizes are presented for descriptive purposes only. Although small patterns of difference were observed in this single-site pilot study, they should not be interpreted as evidence of intervention effects.

The literature emphasizes that hypothesis testing should not rely solely on statistical significance (Ledesma et al., 2008). Effect size estimates provide additional information regarding the magnitude of observed effects and their potential practical relevance (Cohen, 1988). However, in the context of this small pilot study, effect sizes should be interpreted descriptively and not as evidence of practical significance. In the present study, effect sizes were *d* = 0.51 for trait emotional intelligence, *d* = 0.20 for civic attitudes, and *d* = 0.38 for school belonging. These findings are broadly consistent with prior research on service-learning, as reported in systematic reviews (Conway et al., 2009; Filges et al., 2022). Although effect sizes of approximately 0.30 are sometimes considered practically meaningful in educational research (Hattie, 2009), the estimates observed here should be interpreted cautiously as exploratory indicators of potential developmental relevance rather than as evidence of intervention effectiveness.

However, the findings for civic behavior and learning mindset were unexpected, as the control group scored higher than the intervention group. Similar patterns have been reported in early-stage experiential learning interventions. Service-learning often requires students to engage with unfamiliar tasks, increased responsibility, and real-world challenges, which may temporarily reduce perceived competence or confidence before subsequent improvements emerge. These patterns may also reflect a response-shift or calibration effect (Waterman, 2003), whereby students adjust their internal standards after engaging in authentic community-based activities. Self-report measures are particularly sensitive to such changes and may indicate decreases in perceived ability even when actual competence is improving. From a methodological perspective, the cognitively and emotionally demanding nature of the intervention (e.g., collaboration with nongovernmental organizations, environmental fieldwork, and community engagement) may have imposed greater demands on students than the routine academic activities experienced by the control group, which should be considered when interpreting these results. Although these potential mechanisms were not directly examined in the present study, future research should investigate them explicitly, for example, by incorporating alternative measures of the same constructs and including direct assessments of cognitive load and response-shift effects.

This study constitutes a pilot investigation and contributes to the limited quasi-experimental evidence on service-learning in Spanish secondary education (Fernández-Martín & Ayllón-Salas, 2026). Although preliminary, the findings provide insights that may inform future refinements in the design, duration, and implementation of service-learning interventions. The observed patterns may also be related to broader cultural characteristics, such as aspects of Spain’s collectivist orientation, which may influence adolescents’ civic engagement responses (Hofstede, 2001). However, this possibility was not examined in the present study; therefore, replication in culturally diverse contexts is warranted.

Although quasi-experimental designs represent a feasible and valuable methodological approach in educational research, they also present notable challenges. The use of a single intervention group and a single control group may have influenced the results. In addition, the relatively small sample size limits the ability to draw robust conclusions. The use of a nonprobability sampling strategy also constrains the representativeness of the sample and the generalizability of the findings. Nevertheless, these limitations are common in educational research and often reflect practical constraints, including limited school participation due to resource availability, time demands, and institutional considerations.

Several limitations should be acknowledged. The study was conducted in a single school, with only one classroom assigned to each condition, resulting in complete confounding between classroom and treatment assignment (Spybrook et al., 2020). Although pretest-adjusted ANCOVAs reduce baseline nonequivalence, they cannot account for unobserved classroom-level influences. Therefore, the findings should be interpreted as adjusted associations rather than causal effects. Additional limitations include the small sample size, the use of a nonprobability sampling strategy, and reliance on self-report measures, all of which may introduce bias and limit generalizability. Future research should (a) employ designs with multiple classrooms per condition, preferably multisite cluster randomized trials or larger quasi-experimental studies, to disentangle treatment effects from classroom-level variance and strengthen internal validity; (b) increase sample size; (c) secure sufficient resources to support high-quality implementation; (d) use probability sampling methods to enhance generalizability; (e) control for additional variables that may influence intervention outcomes; and (f) incorporate behavioral or observational measures to complement self-report data.

## 5. Conclusions

This pilot study did not provide statistically significant evidence that the service-learning intervention improved trait emotional intelligence or the other outcomes examined. The observed effect sizes across variables should be interpreted as preliminary and exploratory rather than as evidence of intervention efficacy. Nevertheless, the study contributes to the literature by providing an initial evaluation of the PLACE model in Spanish secondary education and by identifying key methodological considerations for future research.

Future studies employing multi-classroom and multisite designs are needed to determine whether the observed patterns can be replicated under more rigorous conditions and to strengthen internal validity. The limitations identified in the present study offer guidance for methodological refinement, underscoring the need for more robust research on service-learning interventions in this population.

## Figures and Tables

**Table 1:** Baseline Characteristics according to Treatment or Control Status.

Variables		Comparison	*M*	*SD*	*d*
Global trait emotional intelligence		Intervention	4.86	0.66	−0.29
		Control	5.06	0.74	
Civic competence					
	Civic attitudes	Intervention	5.16	0.82	−0.05
		Control	5.21	0.82	
	Civic behavior	Intervention	5.50	0.78	0.14
		Control	5.35	1.20	
Academic motivation and engagement					
	Academic perseverance	Intervention	3.93	0.77	−0.13
		Control	4.04	0.84	
	Learning mindset	Intervention	4.09	0.67	0.05
		Control	4.06	0.74	
	School belonging	Intervention	3.83	0.62	0.01
		Control	3.82	0.74	
School dropout intention		Intervention	1.47	1.00	0.02
		Control	1.44	1.01	
Grade point average		Intervention	7.74	1.51	−0.09
		Control	7.89	1.80	

Note. *M* = mean; *SD* = standard deviation; *d* = Cohen’s *d* effect size calculated on pretest scores.

**Table 2:** ANCOVA Results for Global Trait Emotional Intelligence, Civic Competence, Academic Motivation, and Engagement, School Dropout Intention, and Grade Point Average.

Variables			Sum of Squares	df	Mean Square	F	Partial η^2^
Global trait emotional intelligence		Covariate (Pretest)	14.70	1	14.70	103.30 ***	0.66
		Group	0.48	1	0.48	3.43	0.06
		Gender	0.23	1	0.23	1.64	0.20
		Error	7.54	53	0.14		
Civic competence							
	Civic attitudes	Covariate (Pretest)	11.29	1	11.29	19.28 ***	0.27
		Group	0.30	1	0.30	0.52	0.01
		Gender	0.32	1	0.32	0.54	0.01
		Error	31.03	53	0.58		
	Civic behavior	Covariate (Pretest)	5.88	1	5.88	9.08 *	0.14
		Group	2.54	1	2.54	3.93	0.06
		Gender	0.63	1	0.63	0.98	0.02
		Error	34.31	53	0.64		
Academic motivation and engagement							
	Academic perseverance	Covariate (Pretest)	26.68	1	26.68	64.38 ***	0.55
		Group	0.03	1	0.03	0.08	0.00
		Gender	0.10	1	0.10	0.24	0.00
		Error	21.96	53	0.41		
	Learning mindset	Covariate (Pretest)	8.93	1	8.93	41.09 ***	0.43
		Group	0.55	1	0.55	2.54	0.04
		Gender	0.26	1	0.26	1.20	0.22
		Error	11.52	53	0.22		
	School belonging	Covariate (Pretest)	9.90	1	9.90	28.05 ***	0.34
		Group	0.68	1	0.68	1.95	0.03
		Gender	0.36	1	0.36	1.02	0.01
		Error	18.71	53	0.35		
School dropout intention		Covariate (Pretest)	19.41	1	19.41	18.08 ***	0.25
		Group	0.67	1	0.67	0.62	0.01
		Gender	0.23	1	0.23	0.22	0.00
		Error	56.91	53	1.07		
Grade point average		Covariate (Pretest)	86.51	1	86.51	86.92 ***	0.63
		Group	0.28	1	0.28	0.28	0.01
		Gender	0.04	1	0.04	0.04	0.00
		Error	50.75	51	0.99		

Note. * *p* < .05, *** *p* < .001.

**Table 3:** Bonferroni Post hoc Results for Global Trait Emotional Intelligence, Civic Competence, Academic Motivation and Engagement, School Dropout Intention and Grade Point Average.

Variables		Comparison	M	Adj. M. Diff.	Cohen’s *d*	*p*	CI
Global trait emotional intelligence							
		Intervention	4.98	0.19 (0.10)	0.51	.11	−0.11–0.98
		Control	4.79				
Civic competence							
	Civic attitudes						
		Intervention	5.20	0.15 (0.20)	0.20	.36	−0.29–0.78
		Control	5.05				
	Civic behavior						
		Intervention	5.27	−0.43 (0.22)	−0.54	.07	−1.02–0.06
		Control	5.71				
Academic motivation and engagement							
	Academic perseverance						
		Intervention	3.62	0.05 (0.17)	0.08	.85	−0.48–0.58
		Control	3.57				
	Learning mindset						
		Intervention	3.89	−0.20 (0.12)	−0.43	.17	−0.90–0.17
		Control	4.10				
	School belonging						
		Intervention	3.75	0.22 (0.16)	0.38	.24	−0.22–0.85
		Control	3.53				
School dropout intention							
		Intervention	1.48	−0.22 (0.28)	−0.22	.36	−0.78–0.28
		Control	1.71				
Grade point average							
		Intervention	7.41	−0.14 (0.27)	−0.14	.59	−0.68–0.39
		Control	7.56				

Note. Standard errors appearing in parentheses. M = estimated marginal means; Adj. M. Diff. = Adjusted Mean Difference; CI = Confidence Interval.

## Data Availability

Access to data and study materials is available upon request to the authors.

## Appendix A

**Table A1:** Baseline Characteristics by Treatment or Control Status.

Variables		Comparison	N	Sk (SE)	Ku (SE)
Global trait emotional intelligence					
		Intervention	30	−0.51 (0.43)	−0.13 (0.83)
		Control	27	−0.45 (0.45)	0.08 (0.87)
Civic competence					
	Civic attitudes				
		Intervention	30	0.88 (0.43)	0.25 (0.83)
		Control	27	−0.22 (0.45)	−0.99 (0.87)
	Civic behavior				
		Intervention	30	−0.41 (0.43)	−0.58 (0.83)
		Control	27	−1.75 (0.45)	3.94 (0.87)
Academic motivation and engagement					
	Academic perseverance				
		Intervention	30	−0.02 (0.43)	−1.28 (0.83)
		Control	27	−0.71 (0.45)	−0.40 (0.87)
	Learning mindset				
		Intervention	30	−1.08 (0.43)	1.57 (0.83)
		Control	27	−1.51 (0.45)	2.94 (0.87)
	School belonging				
		Intervention	30	−1.15 (0.43)	1.58 (0.83)
		Control	27	−0.26 (0.45)	−0.89 (0.87)
School dropout intention					
		Intervention	30	−2.37 (0.43)	5.27 (0.83)
		Control	27	2.32 (0.45)	5.10 (0.87)
Grade point average					
		Intervention	30	0.17 (0.44)	−1.14 (0.87)
		Control	27	−1.59 (0.44)	3.72 (0.85)

Note. Sk: Skewness, SE: Standard error, Ku: Kurtosis.
